# Methods of treatment in the inequality of legs in children

**Published:** 2012-06-18

**Authors:** R Trancă

**Affiliations:** Carol Davila” University of Medicine and Pharmacy, Bucharest “Grigore Alexandrescu” Emergency Children’s Clinical Hospital, Bucharest

**Keywords:** leg equin, legs inequality, femoral hypoplasia

## Abstract

The medical and biological long-term research led to the discovery of some general laws that govern the stimulation of the tissue growth and the regeneration, and this is called: the “tension stress” law.

It was found that a gradual tension of a living tissue creates a stress what can stimulate and maintain the regeneration of the active growth of that specific tissue. A slow traction, the constant of a tissue, will make it become metabolically active, resulting in an increase of the proliferative and biosynthetic functions. These processes are so dependent on an adequate blood flow at the tissue that it is elongated, but they are also dependant on the stimulatory and functional effect of the carrying weight.

The clinical application of this biological law allowed, for the first time, the control of the healing process and the remodeling of the bone and the soft tissue, which allows the development of some new methods of treatment of the diseases caused by the inequalities of legs

## Introduction

The legs inequality in children represents a pathological condition that is more frequent than it looks at first sight, being caused by a wide variety of medical causes. The little, asymptomatic inequalities, between 5 mm and 2 cm are frequently met, being diagnosed by the physician while presenting to the doctor’s for another medical condition or when the young boys go to the doctor to do the screenings in order to be recruited in the army [**5**].

The therapeutic attitude to an inequality is often controversial and debated by the orthopedic specialists; this being variable according to the size of the inequality, its etiology or the age of the patient. Normally, the shortening of a leg, which is of less than 2 cm during the age of the skeletal maturity, is considered unimportant, no treatment being applied [**4,5**]. However, there is no scientific argument to support this evaluation. 

There are some controversies regarding the correction modality choice, according to the size of the inequality. A draft of the therapeutic attitude looks like this: 

1. Inequalities less than 2 cm: 

• Do not need treatment or prosthesis. 

2. Inequalities between 2 and 5 cm: 

• -The shortening of the healthy leg 

• -The elongation of the shorter leg (acute elongation) 

3. Inequalities between 5 and 15 cm: 

• The elongation of the shorter leg by gradual techniques 

4. Inequality which is more than 15 cm: 

• Prosthesis [**4,5**]

## Materials and methods

The study was done on 12 children, who presented to “Grigore Alexandrescu” Emergency Children’s Clinical Hospital, during 1993-1998 with the diagnostic of legs inequality. 

The causes that led to this condition were various. Three children of the total of 12 had sequelae from hip neonatal osteoarthritis, two of them being diagnosed with bone tumors (a surgery undergone to save the leg), two patients had sequelae from the neurological varus equin leg, five patients suffered from congenital hypoplasia of the femur and two patients suffered from aseptic pseudarthrosis. Surgical treatment was applied to nine children from the group of 12 by using the external fixation, in the other three children, this intervention not being suitable due to general causes. The medium age for the application of this surgical technique was of 12,8 years; between 8 and 17 years old. 

### Surgical technique

The elongation of the shorter leg was obtained in three cases by corticomy and osteoclasy at a certain level. The objective of the surgery, done through a 4-5 mm incision, is that of preserving the bone marrow, the related artery and its branches, as well as the osteogenetic elements in the peribone soft tissues. The surgical technique was combined with the external fixation of the bone fragments in order to insure the necessary conditions for a rapid osteogenesis. Thanks to the minimal surgery, the precocious mobilization of the patient was possible, in order to preserve the joint mobility. In the case of a 12-year-old patient, with a shortening of the right femur due to a newborn osteomyelitis (right coxofemoral osteoarthritis), the translation of the femur to the inferior margin of the cotyloid cavity was practiced. Simultaneously, the femur was elongated in its medium third. In the case of the patients with aseptic pseudarthrosis, the etiology was represented by fractures that were operated on, with the lack of bone consolidation on a period of 10 months to 1 year after the surgery. The osteosynthesis by simple compression and the external bone transport were used as treatment [**1,2**].

The results were good; radiologic check-ups were done from time to time to verify the accuracy of the surgery. Osteotomy was practiced by monofocal compression in three patients. This method implied the concentration in only one source of corticomy or osteotomy. After a latent period, during which the fibrous callus has formed, the gradual removal of the distal fragment with 1 mm on a 24 hours period has allowed the regeneration of the bone, the elongation with 5 cm being realized in a patient and with 7 cm and 8,5 cm in the other two patients [**1,2**].

## Results

The inequality prediction was done at the end of the bone growth in six patients out of the total of 9, with ages under 14 years, in whom the distraction intervention was used. The coefficient method, described by Paley was used because the chronological age should be taken into consideration. Femur bone elongations between 5 and 10 cm and tibia bone elongations between 5 and 7 cm were obtained. The radiologic follow-up of the evolution of the bone regeneration in the patients treated and their control in time have shown a great variety of normal and pathological aspects. The number of days the fixative was worn was, in most of the cases, bigger than necessary. Although the signs of radiologic ossification were present, the external fixative was kept for a longer period for safety reasons and for the success of the treatment. 

## Conclusions

As a result of the legs elongations realized in our clinic, the following conclusions have been reached: 

• The elongations up to 5 cm produce rapid consolidations and do not give complications;

• The elongations of over 5 cm obligatorily need a good psychological preparation and a careful observation [**3**];

• The preoperative clinical examination of the patients is extremely important in order to distinguish between a structural inequality and a possible functional discrepancy of length [**2,3**].

**Fig. 1:**
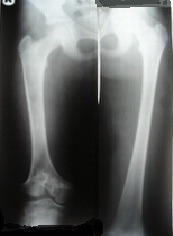
Front X-ray of both femurs before the elongation

**Fig. 2:**
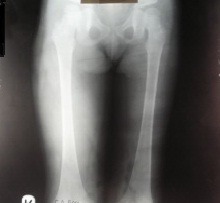
Front X-ray of both femurs at the end of the elongation

**Fig. 3:**
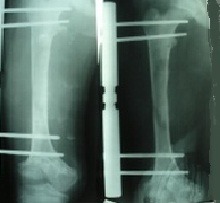
X-ray of the right femur while the 2 cm elongation takes place

**Fig. 4:**
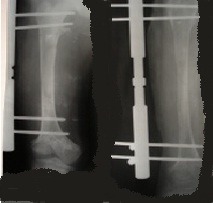
X-ray of the right femur while the 6 cm elongation takes place

**Fig. 5:**
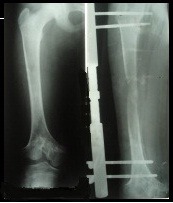
X-ray of the right femur at the end of the 10 cm elongation

The following figures (**Fig. 1-5**) present the radiological aspects of a right leg inequality, elongated through the method of distraction with an external fixative; the elongation being of 10 cm.
